# Heart Rate Variability as a Possible Predictive Marker for Acute Inflammatory Response in COVID-19 Patients

**DOI:** 10.1093/milmed/usaa405

**Published:** 2021-01-30

**Authors:** Frederick Hasty, Guillermo García, Héctor Dávila, S Howard Wittels, Stephanie Hendricks, Stephanie Chong

**Affiliations:** Attending Anesthesiologist, Miami Beach Anesthesiology Associates, Inc., Division of Anesthesiology, Mount Sinai Medical Center, Miami Beach, FL 33140, USA; Clinical Associate Professor of Anesthesiology, Anesthesiology Residency Program, Division of Anesthesiology, Mount Sinai Medical Center, Miami Beach, FL 33140, USA; Clinical Associate Professor of Anesthesiology, Herbert Wertheim College of Medicine, Florida International University, Miami, FL 33199, USA; Clinical Associate Professor of Anesthesiology, Nurse Anesthesia Graduate Programs, Florida International University, University of Miami and Barry University, Miami, FL 33199, USA; Attending Anesthesiologist, Miami Beach Anesthesiology Associates, Inc., Division of Anesthesiology, Mount Sinai Medical Center, Miami Beach, FL 33140, USA; Clinical Associate Professor of Anesthesiology, Anesthesiology Residency Program, Division of Anesthesiology, Mount Sinai Medical Center, Miami Beach, FL 33140, USA; Clinical Associate Professor of Anesthesiology, Herbert Wertheim College of Medicine, Florida International University, Miami, FL 33199, USA; Clinical Associate Professor of Anesthesiology, Nurse Anesthesia Graduate Programs, Florida International University, University of Miami and Barry University, Miami, FL 33199, USA; Attending Anesthesiologist, Miami Beach Anesthesiology Associates, Inc., Division of Anesthesiology, Mount Sinai Medical Center, Miami Beach, FL 33140, USA; Clinical Associate Professor of Anesthesiology, Anesthesiology Residency Program, Division of Anesthesiology, Mount Sinai Medical Center, Miami Beach, FL 33140, USA; Clinical Associate Professor of Anesthesiology, Herbert Wertheim College of Medicine, Florida International University, Miami, FL 33199, USA; Clinical Associate Professor of Anesthesiology, Nurse Anesthesia Graduate Programs, Florida International University, University of Miami and Barry University, Miami, FL 33199, USA; Attending Anesthesiologist, Miami Beach Anesthesiology Associates, Inc., Division of Anesthesiology, Mount Sinai Medical Center, Miami Beach, FL 33140, USA; Clinical Associate Professor of Anesthesiology, Anesthesiology Residency Program, Division of Anesthesiology, Mount Sinai Medical Center, Miami Beach, FL 33140, USA; Clinical Associate Professor of Anesthesiology, Herbert Wertheim College of Medicine, Florida International University, Miami, FL 33199, USA; Clinical Associate Professor of Anesthesiology, Nurse Anesthesia Graduate Programs, Florida International University, University of Miami and Barry University, Miami, FL 33199, USA; Clinical Associate Professor of Anesthesiology, Nurse Anesthesia Graduate Programs, Florida International University, University of Miami and Barry University, Miami, FL 33199, USA; Nurse Anesthetist, Miami Beach Anesthesiology Associates, Inc., Mount Sinai Medical Center, Miami Beach, FL 33140, USA

## Abstract

**Introduction:**

Increases in C-reactive protein (CRP) are used to track the inflammatory process of COVID-19 and are associated with disease state progression. Decreases in heart rate variability (HRV) correlate with worsening of disease states. This observational study tracks changes in HRV relative to changes in CRP in COVID-19 patients.

**Materials and Methods:**

In accordance with an Institutional Review Board-approved study, 17 patients were followed using the wearable, noninvasive Tiger Tech Warfighter Monitor (WFM) that records HRV from a single limb electrocardiogram. Intermittent, daily short-segment data sets of 5 to 7 minutes over a minimum of 7 days were analyzed. Changes in HRV were compared to changes in CRP.

**Results:**

Decreases in HRV of greater than 40% preceded a 50% increase in CRP during the ensuing 72 hours in 10 of the 12 patients who experienced a dramatic rise in CRP. The effectiveness of HRV as a leading indicator of a rise in CRP was evaluated; the sensitivity, specificity, positive predictive value, and negative predictive value for 40% decreases in HRV preceding 50% increases in CRP were 83.3%, 75%, 90.9%, and 60%, respectively.

**Conclusion:**

Substantial decreases in HRV preceded elevations in CRP in the ensuing 72 hours with a 90.9% positive predictive value. Early detection of increasing inflammation may prove vital in mitigating the deleterious effects of an abnormal inflammatory response, particularly in COVID-19 patients. This capability could have a major impact in triage and care of moderate to severe COVID-19 patients in major medical centers as well as field hospitals. This study demonstrates the potential value of short-segment, intermittent HRV analysis in COVID-19 patients.

## INTRODUCTION

COVID-19 patients requiring admission to an intensive care unit represent the minority of cases but can follow a stormy course caused by a pathological inflammatory response, often referred to as “Cytokine Storm”.^1^ Cytokine storm coincides with a steep rise in inflammatory markers, such as C-reactive protein (CRP). Hence, CRP helps clinicians decide when and in whom pharmacological therapies, such as interleukin-6 inhibitors, might be instituted. However, a drawback to this laboratory test is that it may not prompt clinicians to begin therapy soon enough.

A readily available, noninvasive tool that may provide early warning of impending cytokine storm involves analysis of the cadence of cardiac cycles. Heart rate variability (HRV) is a physiological metric, regulated by the autonomic nervous system (ANS), that has been used for decades to evaluate general well-being in various clinical settings.^2^ Because the ANS responds quickly to changes in physiological states, it may offer signals, in the form of HRV, that can warn of an impending cytokine storm sooner than other currently employed laboratory tests. It is probable that earlier recognition of clinical deterioration, by triggering earlier therapeutic interventions, could better the chance of positive outcomes. However, the ability to extract, analyze, and calculate HRV from our standard hospital monitors was impossible for this study. Our standard hospital monitors are not technically set-up for HRV analysis because of the challenge of extracting the necessary electrocardiogram (ECG) data for HRV calculations.

The relation between HRV and inflammatory states has been extensively studied. A recent meta-analysis of over 51 studies encompassing a total of 2,238 patients demonstrated an inverse relationship between HRV and inflammation.^3^ However, a precise mechanism for how the immune system and the ANS interact to impact the HRV has not been described.^4^ A common measure of HRV is the standard deviation of the interval between heartbeats (SDNN). This measure of HRV has been shown to correlate inversely with the nonspecific inflammatory marker CRP.^4^

In the current COVID-19 pandemic, CRP has become vital to monitoring patients’ inflammatory status.^5^ Clinicians across the globe are using CRP to stratify and predict disease severity in COVID-19.^6^ Large rises in CRP and interleukin-6 coincide with cytokine storm and with the progression to more severe disease states.^1^ Interleukin-6 levels, while more specific to cytokine storm,^7^ often require blood samples to be sent to specialized laboratories, resulting in extended turn-around times for results. C-reactive protein levels, on the other hand, are readily available for most hospital clinicians. Therefore, physicians in the community follow CRP values to determine the onset of this hyper-inflammatory state. Current literature has found elevated CRP to be an independent factor predicting disease risk and correlating with disease severity.^5,6^ In the present study, we set out to determine whether decreases in HRV predict elevations in CRP in COVID-19 patients.

## METHODS

### Patients

After obtaining approval and waiver of consent from Mount Sinai Medical Center Institutional Review Board, we recruited patients admitted to this facility from April 9 through May 8, 2020. Inclusion criteria were patients 18 years or older with polymerase chain reaction-positive nasopharyngeal swabs for SARS-CoV-2 admitted to the COVID ICU and step-down units for a minimum of 7 days. All patients presented with hypoxic respiratory failure requiring high-flow nasal cannula or mechanical ventilation. Excluded patients included those who refused to participate, were in roto-beds, on extracorporeal membrane oxygenation, or in hospice. Seventeen patients were enrolled as a random convenience sample. These were nonsequential patients. Measurements of HRV and CRP were made on each patient over a minimum of 7 days to provide sufficient time to observe trends. Patient demographic data and comorbidities are summarized in Table I.

**TABLE I.** Patient Demographics and Characteristics

Mean age (SD) and range	60.5 years (13.4) 31–85		Comorbidities	
Sex	71%	Male	71%	Cardiovascular disease
Ethnicity	53%	Hispanic	24%	Pulmonary disease
	29%	African American	35%	Renal disease
	6%	Asian American	6%	Immunocompromised
	12%	Caucasian	12%	Previous or current cancer
Mean BMI (SD) and range	32.1 (6.5) 23–42		29%	Diabetic

### Data Collection

**FIGURE 1.**
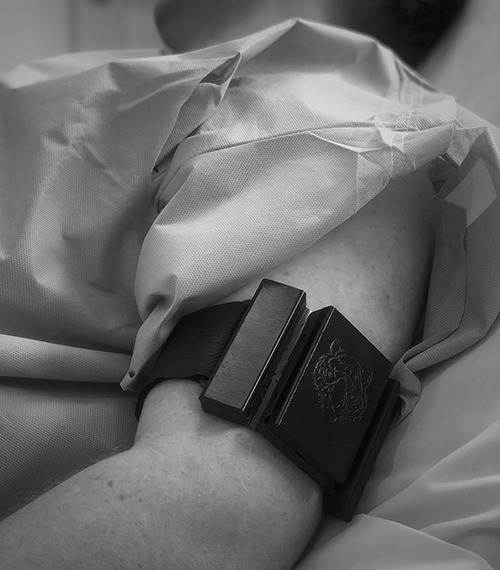
The Warfighter Monitor measures heart rate and heart rate variability from a single limb electrocardiogram.

The research personnel donned all appropriate personal protective equipment throughout the time they were in the patient’s room. A wearable monitoring device was used to measure heart rate and HRV from a single limb ECG attributable to the lack of HRV analysis capabilities of our standard ICU monitors. The device selected for data collection was the Tiger Tech Warfighter Monitor (Miami, FL)^8^ as shown in Fig. 1. As a result of limitations of resources and exposure risk, short-segment HRV data sets were collected over 7 minutes (a time window sufficient to gather ECG baseline readings and to analyze HRV).^9^ Measurements were taken in the morning to eliminate diurnal variation. A designated, secure phone received and archived the data from the Warfighter Monitor via Bluetooth. When not in use for the study, the phone was kept in a secured site on Mount Sinai Medical Center campus. Heart rate variability was calculated using the standard deviation of the NN interval (SDNN).

A large, 3M Tegaderm (St. Paul, MN) was placed on the lateral aspect of the bicep to prevent contamination of the device. The device was then placed on top of the Tegaderm and secured to the patient with a second Tegaderm. At the completion of the 7-minute reading, the device was removed from the patient and thoroughly decontaminated with 70% Alcohol Metrex CaviWipes (Orange, CA). The Tegaderm was removed from the patient and the researchers doffed their personal protective equipment.

## RESULTS

**FIGURE 2.**
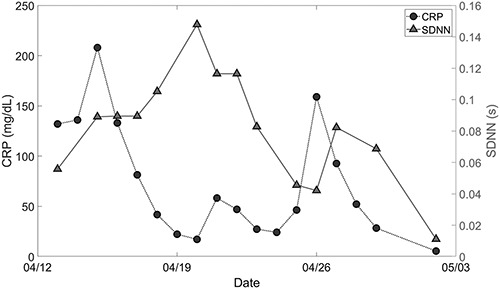
An example of one patient’s biometrics over time. The charted lines represent heart rate variability in a triangulated line and C-reactive protein (CRP) in a blocked line. A drop of more than 40% in standard deviation of the interval between heartbeats (SDNN) is followed by more than a tripling of CRP in the subsequent 72 hours.

Of the 17 patients studied, data from one patient were too inconsistent to analyze and are not discussed further here. Of the remaining 16 patients, 12 developed a greater than 50% increase in CRP during the study period. Of the 12 patients who developed a greater than 50% increase in CRP, 10 demonstrated a greater than 40% drop in HRV within a 72-hour window preceding the increase in CRP. An example of the temporal relationship between HRV and CRP recorded from one patient over several days is shown in Fig. 2. Fig. 2 plots on the same abscissa CRP as mg/dL with respect to the left ordinate and HRV as SDNN with respect to the right ordinate.

The data collection in Fig. 2 begins on April 13. A characteristic decrease in HRV is demonstrated from April 20 to April 23 which is followed by the rise in CRP from April 23 to April 26. This is representative of the findings in 10 of the 12 patients who had an increase in CRP. As with all of the patients studied, the relationship between HRV and CRP at the beginning and end of the studies could not be completely analyzed because the preceding HRV and subsequent CRP levels were not recorded. However, during the study period, we consistently found a correlation between a >40% decrease in HRV and subsequent 50% rise in CRP.

The consistency of the relation between the drop in HRV preceding the rise in CRP is examined in the 2 by 2 contingency matrix presented as Table II. The upper pair of cells in the matrix presents the number of patients with the decrease in HRV of more than 40% while the lower pair of cells presents the number of patients with no such HRV decrease. The left half of the table presents the number of patients in which CRP increased by more than 50% while the right half of the table presents the number of patients in which no such increase occurred.

**TABLE II.** Calculations of Sensitivity, Specificity, Positive Predictive Value, and Negative Predictive Value

	CRP increases > 50%	CRP does not change	
HRV	True positive (TP)*^a^*	False positives (FP)*^b^*	Positive predictive value
decreases	10	1	90.9% [=10/11*100]
>40%			
HRV	False negatives (FN)*^c^*	True negatives (TN)*^d^*	Negative predictive value
unchanged	2	3	60% [=3/5*100]
	Sensitivity	Specificity	
	83.3% [=10/12*100]	75% [=3/4*100]	

a ↓HRV 40% followed by ↑CRP 50% (within 72 hours).

b ↓HRV > 40% without ↑CRP.

c ↑CRP without a >40% ↓HRV.

d No ↓HRV 40% nor ↑CRP.

From this table, it is possible to assess the usefulness of HRV as a leading indicator for an increase in CRP, at least for the present dataset. Thus, the number of HRV true positive over the total number of CRP increases showed that HRV had a calculated leading indicator sensitivity of 83.3%. Moreover, HRV is specific to the CRP increase since the HRV decrease did not occur without a subsequent CRP increase three out of four times, producing a calculated leading indicator specificity of 75%. Furthermore, from these data, 90.9% of the time the HRV decrease was associated with the CRP increase, termed “positive predictive value” (PPV); whereas 60% of the time the absence of an HRV decrease was associated with the absence of the CRP increase, termed “negative predictive value” (NPV).

## DISCUSSION

In this study, we tracked HRV and CRP changes in 16 COVID-19 patients. In this small series, 75% of patients experienced a dramatic rise in CRP during the study period. In those patients, we found a threshold decrease (>40%) in HRV to be 83.3% sensitive and 75% specific for subsequent significant rises in CRP. Furthermore, our PPV and NPV were 90.9% and 60%, respectively. This threshold yielded the optimum PPV and NPV while maintaining the highest sensitivity and specificity. We did not find a similar relationship when using absolute values for changes in HRV. Further studies are required to explain the temporal relationship between HRV and CRP.

Of the 12 patients who demonstrated large rises in CRP, 2 did not demonstrate decreases in HRV. Of these, one patient received convalescent plasma the day before the surge in CRP (<2.5 to 63 mg/dL in 24 hours). The second patient was in a convalescent stage when her CRP rose >50% in 48 hours while her clinical condition remained unchanged.

In the 10 patients who demonstrated dramatic decreases in HRV with subsequent increases in CRP, the clinical significance varied. All 10 had severe infections with hypoxic respiratory failure and required a minimum of high-flow nasal cannula during the study period. Two did not require intubation. One non-intubated patient who progressed from severe to mild disease demonstrated a large decrease in HRV with a subsequent large increase in CRP which appeared to be of no clinical significance. A second non-intubated patient experienced a decrease in HRV followed by an increase in CRP and transitioned from non-rebreather facemask to high-flow nasal cannula in the prone position. He subsequently recovered after a 2-week stay in the hospital. The eight intubated patients experienced a large decrease in HRV with rises in CRP which corresponded to worsening pulmonary function.

Changes in HRV of >40% carried a 90.9% PPV for increases in CRP. In 10 of the 12 patients who demonstrated an increase in CRP of >50%, the clinical condition of the patients deteriorated. In one of those patients, the HRV decreased in spite of normal vital signs, inflammatory markers, and decreasing O_2_ requirements. In response to this apparent clinical improvement, the primary care team gradually weaned therapy. The patient subsequently deteriorated 72 hours later with massive rises in CRP. She developed severe hypoxemia and required mechanical ventilation. In this case, the drop in HRV warned of deterioration despite the appearance of clinical improvement

Several factors contributed to the limitations of this observational study. There were no controls correlating daily HRV and CRP levels in patients with hypoxic respiratory failure. Frequency of laboratory testing and therapeutic interventions were not standardized because they were ordered at the discretion of the primary care team. Daily critical illness severity scores such as Multiple Organ Dysfunction Score or Sequential Organ Failure Assessment Score would have been beneficial in analyzing the clinical significance of both changes in HRV and CRP. However, the requisite laboratories needed for critical illness severity scores were not universally available every day for each patient. Furthermore, the small sample size limits generalizability. Nonetheless, the results warrant further investigation.

To our knowledge, this is the first study to suggest a clinical value in intermittently assessing HRV in admitted COVID-19 patients. The overall thrust of current efforts to treat this disease focuses on intervening early, particularly in mitigating the sometimes-overwhelming effects of a cytokine storm. Currently, clinicians look for a sharp rise in CRP before instituting anti-inflammatory therapeutics. An earlier prompt to begin pharmacological intervention intuitively would seem advantageous to combatting or even preventing a cytokine storm. Because it can be difficult to predict which patients will need anti-inflammatory interventions, short-segment HRV metrics in COVID-19 patients may prove to be a noninvasive leading indicator aid for triage and subsequent treatment throughout the disease course.

## CONCLUSIONS

Dramatic drops in HRV correlated with subsequent spikes in CRP in COVID-19 patients. Daily short-segment HRV readings may aid clinicians in the triage, disease progress monitoring, and treatment. Intermittent HRV analysis could provide an early warning of an impending inflammatory response (e.g., cytokine storm). Furthermore, this capability could have a major impact in the care of moderate to severe COVID-19 patients in major medical centers, as well as field hospitals. Larger, prospective, randomized, multisite-controlled trials are needed to evaluate the potential value of HRV in the management of COVID-19 patients throughout the course of their hospitalizations.
